# 

**Published:** 1891-07-25

**Authors:** 


					WINdUKNc o
INFANTS
FOR
l5ll*bLAI>i>
AND INVALIDS.
No. 2j52. Vol. X.] Saturday,
July 25th, 1891.
[One Penny.

				

